# Forming a Homeotropic SmA Structure of Liquid Crystalline Epoxy Resin on an Amine-Modified Surface

**DOI:** 10.1021/acsomega.3c01498

**Published:** 2023-08-31

**Authors:** Shingo Tanaka, Yoshitaka Takezawa, Kiyoshi Kanie, Atsushi Muramatsu

**Affiliations:** † Research & Development Group, Hitachi, Ltd., 7-1-1 Omika, Hitachi 319-1292, Ibaraki, Japan; ‡ Advanced Technology Research & Development Center, Showa Denko Materials Co., Ltd., 48 Wadai, Tsukuba 300-4247, Ibaraki, Japan; § Institute of Multidisciplinary Research for Advanced Material, Tohoku University, 2-1-1 Katahira, Aoba-ku, Sendai 980-8577, Miyagi, Japan; ∥ International Center for Synchrotron Radiation Innovation Smart, Tohoku University, 2-1-1 Katahira, Aoba-ku, Sendai 980-8577, Miyagi, Japan

## Abstract

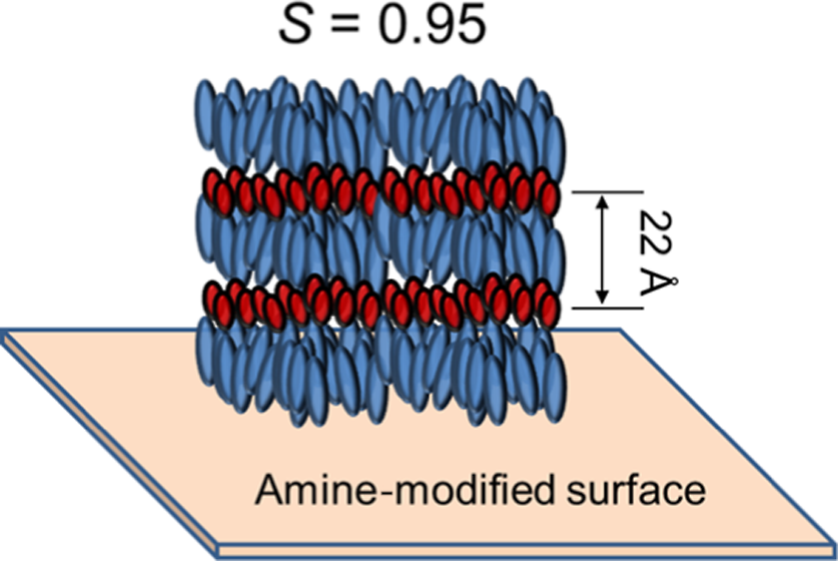

The molecular orientation of a liquid crystalline (LC) epoxy resin (LCER) on silane coupling surfaces of amorphous soda-lime-silica glass substrates was investigated. The LC epoxy monomer on the silane coupling surfaces of the substrates was revealed to form a smectic A (SmA) phase with planar alignments because of the relatively low surface free energy. An LCER with a curing agent, however, formed a homeotropically aligned SmA structure by curing on a substrate surface modified using a silane coupling agent with amino groups. This formation of homeotropic alignment was considered due to the attribution of the reaction between the amino group on the surface of the substrate and the epoxy group of the LCER. The homeotropic alignment had a relatively high orientation parameter of 0.95. Therefore, it is expected to possess high thermal conductivity and be applied as high-thermal-conductivity adhesives or packaging materials for electrical and electronic devices.

## 1 Introduction

Epoxy resin (ER) has been widely adopted to a diverse range of applications because of its thermal resistance, electrical insulation, moldability, and adhesiveness. These properties meet the component requirements of electric and electronic devices. Among ERs, liquid crystalline (LC) ERs have been intensively synthesized^1−10^ and investigated because of their outstanding properties such as high thermal conductivity,^11−22^ fractural toughness,^23−26^ and moisture resistance,^27^ in addition to the properties of general LCs or amorphous ERs. Particularly, the high thermal conductivity of LCERs has gathered attention because electrical and electronic devices progress in downsizing, integrating, and multi-functionalizing, and the heat accumulated in the devices has become a critical issue.

The property of LCERs depend much on their molecular orientation. For instance, the thermal conductivity of an aligned LCER in the direction of the molecular chains is much higher than that in the transverse direction of the molecular chains.^12^ Thus, forming a uniaxial alignment of molecular chains is effective in improving thermal conductivity in the direction of the molecular chains. Although general thermoplastic polymers can be molecularly oriented by stretching, it is difficult for thermosets. Alternatively, applying electric^28^ or magnetic^12,29,30^ fields during cure is effective for a LCER to form a uniaxial alignment.

Whereas a homeotropic alignment for typical LC molecules is generally formed by a surface effect. According to Creagh’s conception,^31^ a homeotropic alignment is induced on the surface of a substrate that possesses lower surface free energy than that of a LC [surface free energy of substrate (γ_S_) < surface free energy of liquid (γ_L_)], and a planar alignment is induced on a higher energy surface (γ_S_ > γ_L_). However, most of these were the results regarding the typical alkyl-terminated LC molecules, and exceptions also occurred.^32^ In our previous study, a LC epoxy monomer was also an exception to Creagh’s conception and homeotropically oriented on a substrate with high γ_S_.^18^ The thermal conductivity of the cross-linked LCER forming a homeotropically aligned smectic A (SmA) structure on a substrate with high γ_S_ was 0.81–5.8 W m^–1^ K^–1^.^18^ From wide-angle X-ray diffraction and grazing-incidence small-angle X-ray scattering (GISAXS) measurements, this outstanding thermal conductivity resulted from the uniaxially aligned SmA structure with 0.70–0.75 of the orientation parameters.^18,19^

Chemical treatment has been widely applied to the ceramic filler surfaces of a composite to increase the adhesiveness of the filler–matrix interfaces and increase the mechanical property,^33−37^ thermal conductivity,^38−41^ and so forth^42−44^ of the composites. However, when chemically treating with a coupling agent to ceramic surfaces, the effect of the functional group on the molecular ordering of a LCER has not been fully elucidated. In this study, we investigated the molecular orientation of a LC epoxy monomer and conventional LC molecules on a ceramic surface modified using silane coupling agents. The LC epoxy monomer was cured with a curing agent on the surface of the substrates modified with the silane coupling agents. The orientational order of the cured LCER on their surfaces was then investigated using GISAXS. As a result, the cured LCER formed homeotropic alignment with 0.95 of the orientation parameter on the ceramic surface modified with the amino group, even though the surface had a low γ_S_.

## 2 Experimental Section

### 2.1 General

Amorphous soda-lime-silica glass substrates (Matsunami Micro Slide Glasses) were used for polarized optical microscope (POM) observations as well as contact angle and GISAXS measurements. Trimethoxy methyl silane (Tokyo Chemical Industry, hereinafter called **C1**), hexyl trimethoxy silane (Tokyo Chemical Industry, hereinafter called **C2**), 3-glycidyloxypropyl trimethoxy silane (Shin-Etsu Chemical, hereinafter called **C3**), trimethoxy [3-(phenylamino) propyl] silane (Shin-Etsu Chemical, hereinafter called **C4**), and 3-(trimethoxysilyl) propylamine (Shin-Etsu Chemical, hereinafter called **C5**) were used to modify the glass substrate surfaces. The water was deionized. Hexadecane (Wako Pure Chemicals, 084-03683) was used as received. Their contact angles were measured using a Kyowa Interface Science CA-D goniometer, applying the sessile drop method.

A tri-cyclic mesogen LC epoxy monomer (Sumitomo Chemical, hereinafter called **TM**(17−19)), 4-cyano-4′-pentylbiphenyl (Sigma-Aldrich, 5CB, hereinafter called **R1**), 4-(*trans*-4-*n*-propylcyclohexyl)-ethoxy-benzene (Merck, ZLI-1476, hereinafter called **R2**), and 1,5-diaminonaphthalene (Sun Chemical, hereinafter called **DAN**) were used as received. The determinations of LC phases were carried out with a Nikon Optiphot-2 Pol. POM is equipped with a Mettler FP82 HT hot stage. Sekisui Chemical Micropearl SP-210 spherical spacers with an average diameter of 10.0 ± 0.5 μm were used to fabricate 10 μm gap glass cells. GISAXS measurements were carried out using a Rigaku Nano-viewer system using Cu Kα radiation (40 kV, 30 mA) equipped with a 5-axis stage. A PILATUS-100K detector was used for the measurements.

### 2.2 Surface Treatments and Estimation of Surface Free Energy of Substrates

**Figure 1:**
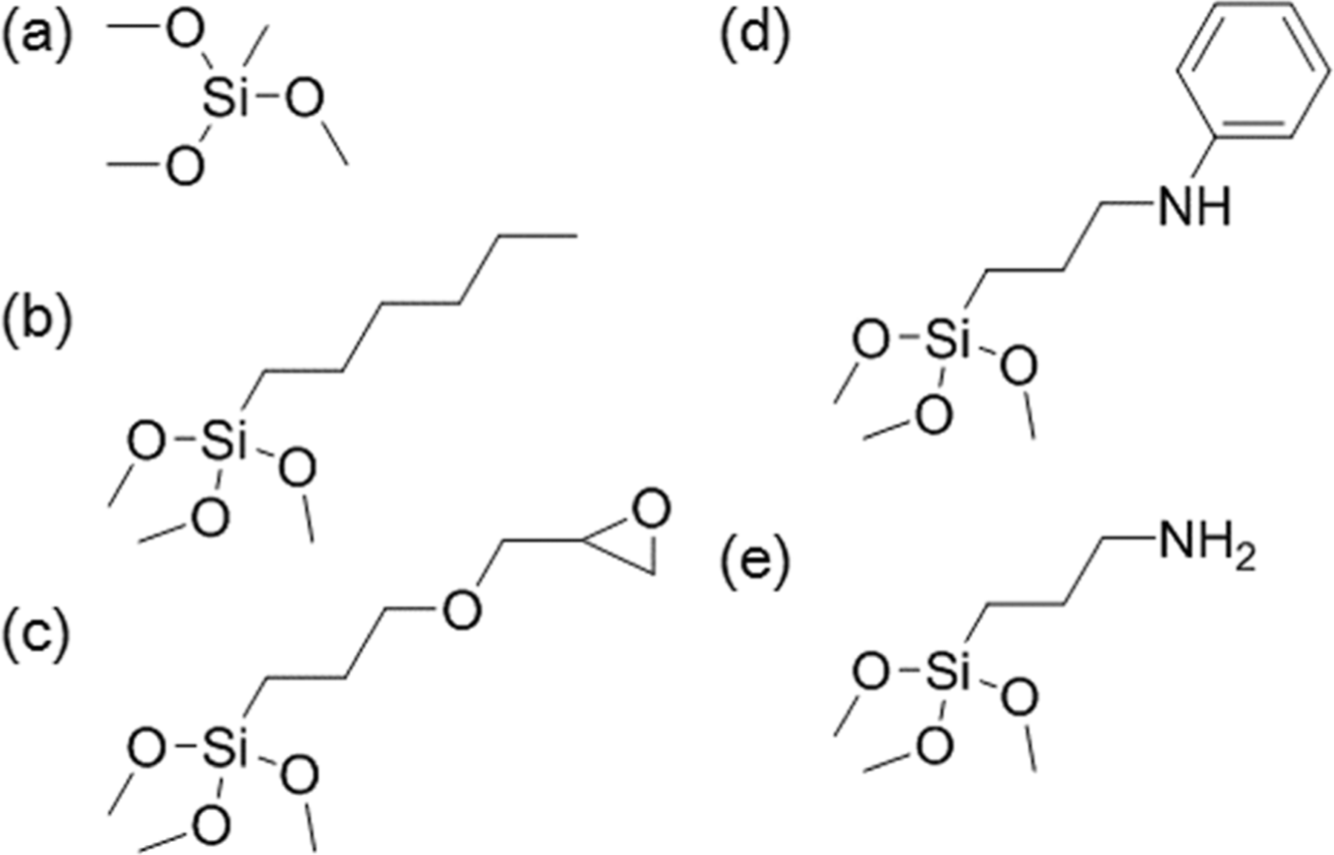
Chemical structures of (a) **C1**, (b) **C2**, (c) **C3**, (d) **C4**, and (e) **C5**.

Amorphous soda-lime-silica glass substrates having six different surface states were prepared. One was untreated (hereinafter called **G1**), and the others were dipped in a 2 wt % **C1**, **C2**, **C3**, **C4**, and **C5** (Figure 1) methanol/water (90/10 w/w) solution and dried at 120 °C for 15 min (hereinafter called **C1**/**G1**, **C2**/**G1**, **C3**/**G1**, **C4**/**G1**, and **C5**/**G1**).

The contact angles of water and hexadecane on **G1**, **C1**/**G1**, **C2**/**G1**, **C3**/**G1**, **C4**/**G1**, and **C5**/**G1** were obtained at ambient temperature with a goniometer applying the sessile drop method. The equilibrium relationship between the vectors of the three-phase interfaces regarding a liquid droplet on a flat solid surface can be described by Young’s equation
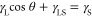
1

Owens–Wendt^45^ further expanded the equation using the terms of the dispersive and polar components of the surface free energy with the geometric mean method
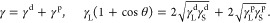
2

The surface free energy components of the liquids are listed in Table 1,^46^ and the resulting contact angles were assigned to eq 2. The surface free energy components of the surfaces of **G1**, **C1**/**G1**, **C2**/**G1**, **C3**/**G1**, **C4**/**G1**, and **C5**/**G1** substrates were calculated using eq 2.

**Table 1:** γ_L_^d^, γ_L_^p^, and γ_L_ of Water, Diiodomethane, and Hexadecane^a^

	surface free energy (mN m^–1^)		
liquid	γ_L_^d^	γ_L_^p^	γ_L_
hexadecane	27.6	0.0	27.6
water	29.1	43.7	72.8

a (γ_L_^p^ and γ_L_^h^ in ref (46) were summarized as γ_L_^p^).

### 2.3 Chemical Structure and LC Behavior of LC Epoxy Monomer and Typical LC Molecules

**Figure 2:**
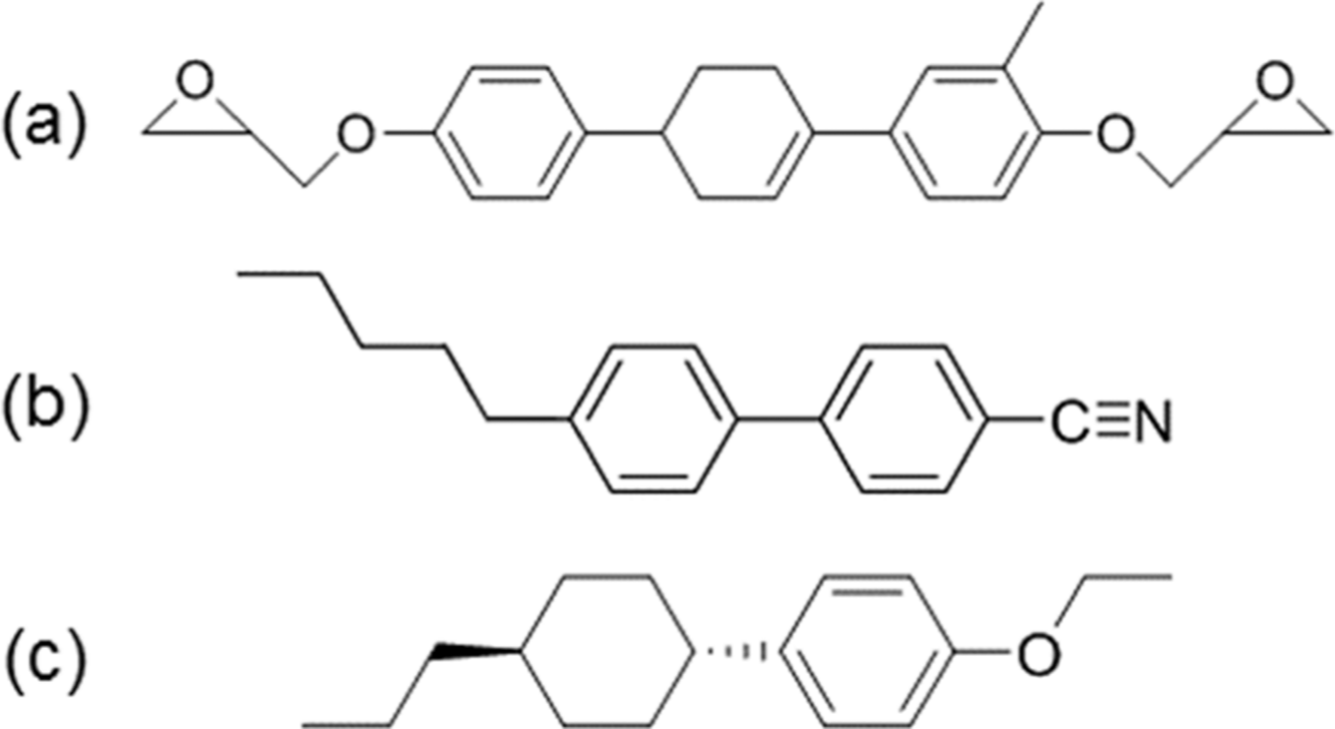
Chemical structures of (a) tri-cyclic-type mesogenic epoxy monomer **TM**, (b) **R1**, and (c) **R2**.

The chemical structures of **TM**, which we used, are shown in Figure 2a. **TM** exhibits a phase sequence on heating the crystalline (Cr) (97 °C) SmA (140 °C) isotropic (Iso) phase and on cooling Iso (139 °C) nematic (N) (138 °C) SmA (50 °C) Cr phase.^17^ Textures of **TM** between pairs of each **G1**, **C1**/**G1**, **C2**/**G1**, **C3**/**G1**, **C4**/**G1**, and **C5**/**G1** with 10 μm gaps were observed using a POM under crossed polarizers at 130 °C.

To investigate the relationship between the polarity of a LC molecule and its orientational order, **R1** and **R2** were also observed with a POM under crossed polarizers on **G1**, **C1**/**G1**, **C2**/**G1**, **C3**/**G1**, **C4**/**G1**, and **C5**/**G1**. The chemical structures of **R1** and **R2** are shown in Figure 2b,c. **TM** possesses polar groups at both ends, **R1** possesses a polar group at the ends of the molecule, and **R2** has no polar groups at the ends of the molecule.

### 2.4 Preparation of TM/DAN Mixtures

The droplets of **TM**/**DAN** mixtures in a 1:1 stoichiometric ratio were cured on **C1**/**G1**, **C2**/**G1**, **C3**/**G1**, **C4**/**G1**, and **C5**/**G1** at 150 °C for 2 h with a thickness of 150–200 μm. GISAXS measurements of the cured droplets were carried out at room temperature. **TM**/**DAN** on **G1** had not been prepared in this study because GISAXS measurements of it had already been reported in our previous paper.^18,19^

## 3 Results and Discussion

### 3.1 Surface Free Energy of Silane-Treated Glass Substrates

**Figure 3:**
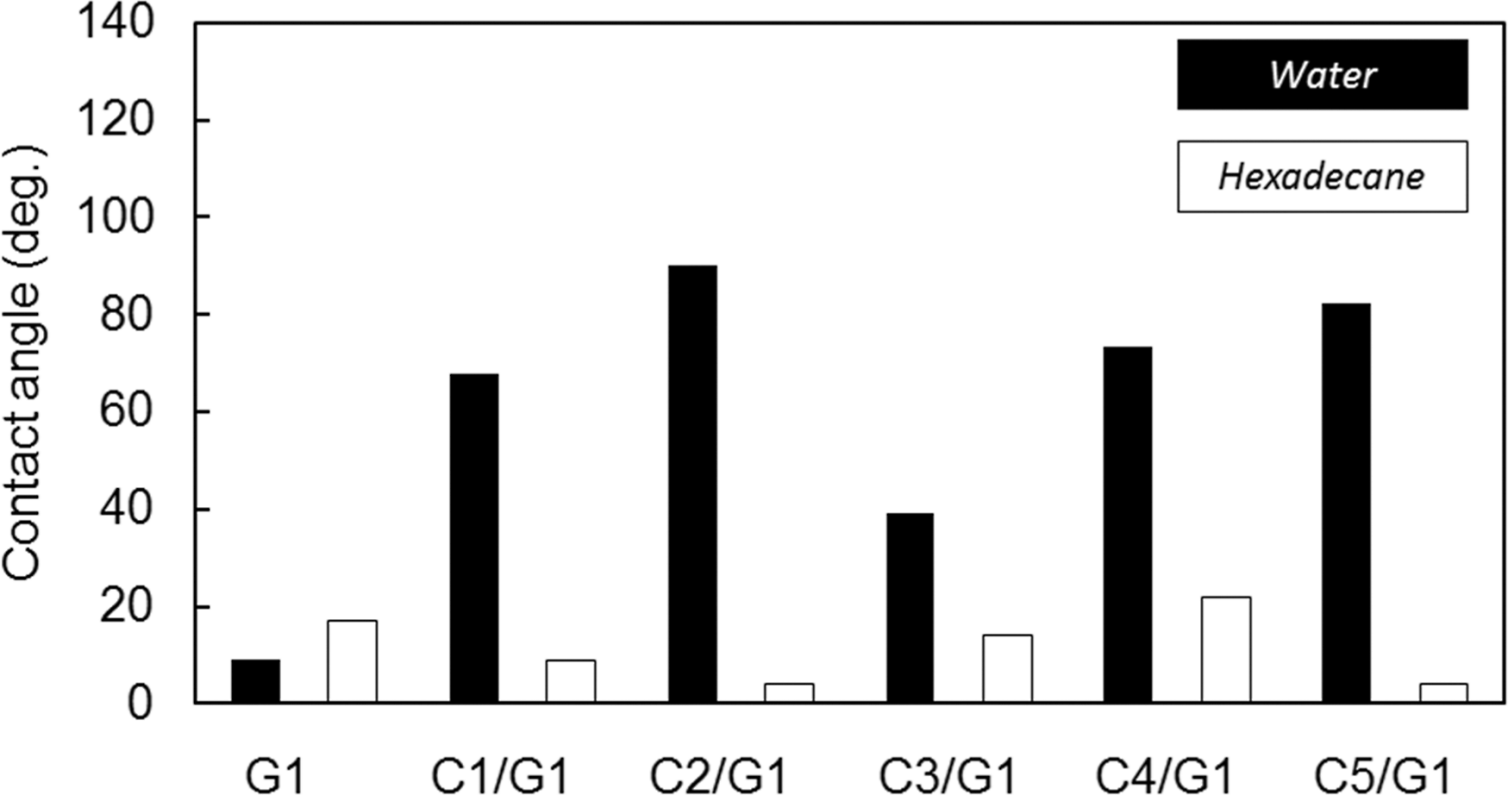
Contact angles of water and hexadecane droplets on **G1**, **C1**/**G1**, **C2**/**G1**, **C3**/**G1**, **C4**/**G1**, and **C5**/**G1**.

The contact angles of water droplets as a polar liquid and hexadecane droplets as a nonpolar liquid on the surfaces of **G1**, **C1**/**G1**, **C2**/**G1**, **C3**/**G1**, **C4**/**G1**, and **C5**/**G1** are shown in Figure 3. The contact angles of water droplets on these surfaces of **C1**/**G1**, **C2**/**G1**, **C3**/**G1**, **C4**/**G1**, and **C5**/**G1** were all higher than those of **G1**. This indicated that **G1** was the most hydrophilic among them. It is also obvious by the comparison of γ_S_^p^ of the substrates in Table 2 estimated using eq 2.

**Table 2:** γ_S_^d^, γ_S_^p^, and γ_S_ of **G1**, **C1**/**G1**, **C2**/**G1**, **C3**/**G1**, **C4**/**G1**, and **C5**/**G1**

	G1	C1/G1	C2/G1	C3/G1	C4/G1	C5/G1
γ_S_^d^	26.4	27.3	27.5	26.8	25.6	27.5
γ_S_^p^	45.6	11.1	1.5	30.8	8.8	3.9
γ_S_	72.0	38.4	29.0	57.6	34.4	31.4

### 3.2 Characteristics of LC Molecules

**Figure 4:**
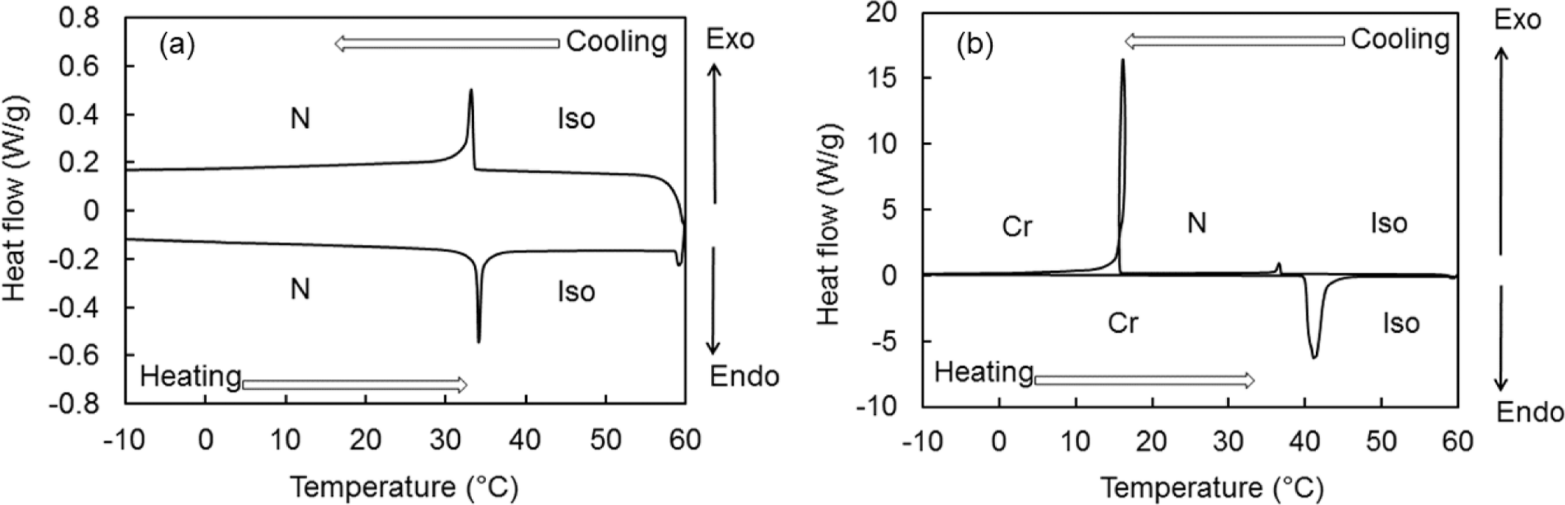
DSC heating and cooling curves of (a) **R1** and (b) **R2** measured at rate of 10 °C/min.

The differential scanning calorimetry (DSC) heating and cooling curves of **R1** and **R2** are shown in Figure 4. The curves of **R1** showed an endothermic peak at 34 °C in the heating trace, derived from the N-Iso transition, and an exothermic peak at 33 °C in the cooling trace. However, **R2** showed a monotropic N phase. An endothermic peak at 41 °C derived from the Cr-Iso transition appeared in the heating trace, while two exothermic peaks at 16 and 37 °C, indicating the Cr–N and N-Iso transitions, respectively, appeared in the cooling trace. The loop-shaped exothermic peak at 16 °C in Figure 4b is thought to be caused by the temperature rise due to the large amount of heat generated with crystallization. DSC curves of **TM** were reported in our previous paper,^17^ and **TM** shows phase sequences on heating Cr (97 °C) SmA (140 °C) Iso phase and on cooling Iso (139 °C) N (138 °C) SmA (50 °C) Cr phase.

### 3.3 Characteristics of LC Molecules between Silane-Treated Glass Substrates

**Figure 5:**
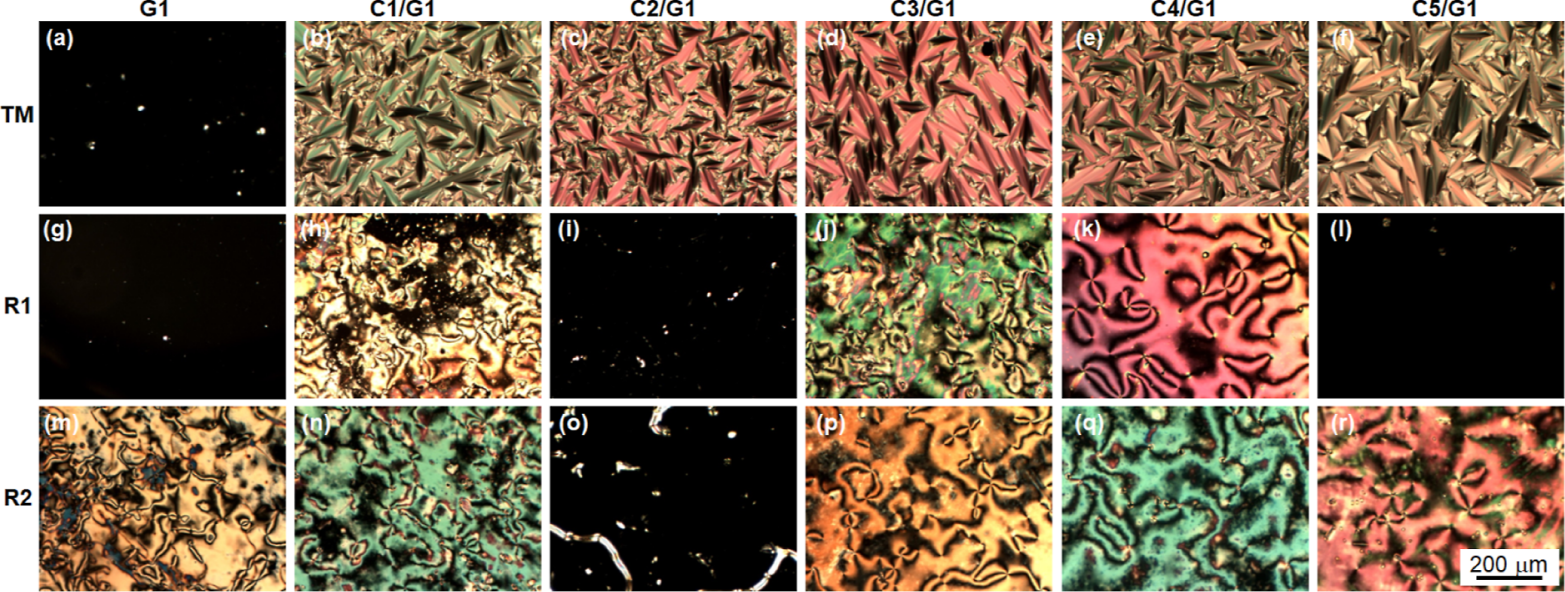
Optical micrographs of **TM** between pairs of each (a) **G1**, (b) **C1/G1**, (c) **C2/G1**, (d) **C3/G1**, (e) **C4/G1**, and (f) **C5/G1** at 130 °C, those of **R1** between pairs of each (g) **G1**, (h) **C1/G1**, (i) **C2/G1**, (j) **C3/G1**, (k) **C4/G1**, and (l) **C5/G1** at 30 °C, and those of **R2** between pairs of each (m) **G1**, (n) **C1/G1**, (o) **C2/G1**, (p) **C3/G1**, (q) **C4/G1**, and (r) **C5/G1** at 30 °C taken under crossed polarizers. Gaps between substrate pairs were 10 μm. Scale bar in (f) is common for all images.

The POM observations under crossed polarizers revealed that **TM** sandwiched between pairs of each **C1**/**G1**, **C2**/**G1**, **C3**/**G1**, **C4**/**G1**, and **C5**/**G1**, which possess relatively low γ_S_, showed a fan-shaped texture of a planar-aligned SmA phase, as shown in Figure 5b–f, while **TM** sandwiched between a pair of **G1** that possesses high γ_S_ showed dark fields at 130 °C after cooling from the Iso phase, as shown in Figure 5a. The dark fields were thought to be derived from a homeotropically aligned SmA phase.^18^ These results were consistent with our previous report, in which homeotropic alignments were induced on high-energy substrates and planar alignments were induced on low-energy substrates.^18,19^ The homeotropically alignments of **TM** were considered to be attributed to the formation of hydrogen bonds between the hydroxyl-terminated surfaces of **G1** and the epoxy groups of **TM**.

The POM regarding **R1**, which possesses a polar group on one side of a molecular termination, sandwiched between pairs of each **C1**/**G1**, **C3**/**G1**, and **C4**/**G1** that possess middle γ_S_, showed schlieren textures of a planar aligned N phase at 30 °C after cooling from the Iso phase, as shown in Figure 5h,j,k, while **R1** sandwiched between pairs of **G1** that possesses the highest γ_S_ and **R1** sandwiched between pairs of each **C2**/**G1** and **C5/G1** that possess relatively low γ_S_ showed dark fields at 30 °C after cooling from the Iso phase, as shown in Figure 5g,i,l. The dark fields were thought to be derived from a homeotropically aligned N phase. The homeotropically alignment N of **R1** on **G1** was considered to be attributed to the polar interaction between the hydroxyl-terminated surfaces of **G1** and the cyano group of **R1**, while the homeotropically aligned N of **R1** on **C2**/**G1** or **C5/G1** was considered to be attributed to the nonpolar interaction between the alkyl surfaces of **C2**/**G1** or **C5/G1** and the terminal alkyl group of **R1**. For a LC molecule with no polar terminations, **R2** sandwiched between pairs of each **G1**, **C1**/**G1**, **C3**/**G1**, **C4**/**G1**, and **C5**/**G1** that possess high γ_S_ showed schlieren textures of a planar aligned N phase, as shown in Figure 5m,n,p–r, while **R2** sandwiched between a pair of **C2**/**G1** that possesses the lowest γ_S_ showed dark fields derived from a homeotropically aligned N phase at 30 °C after cooling from the Iso phase, as shown in Figure 5o. The homeotropic alignment of **R2** on **C2**/**G1** was also considered to be attributed to the nonpolar interaction between the alkyl surfaces of **C2**/**G1** and the terminal alkyl group of **R2**, as well as that of **R1** on **C2**/**G1** or **C5/G1**.

**Figure 6:**
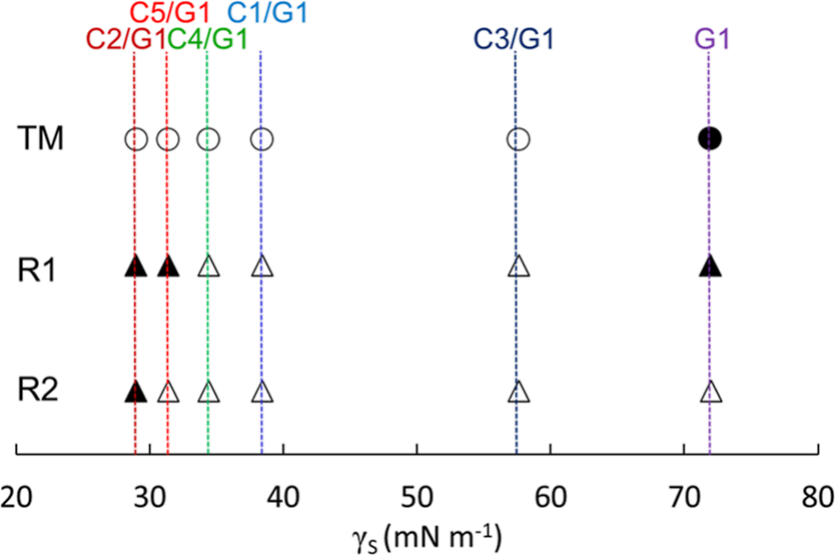
Relationship between γ_S_ and textures of **TM**, **R1**, and **R2** sandwiched between pairs of each **G1**, **C1**/**G1**, **C2**/**G1**, **C3**/**G1**, **C4**/**G1**, and **C5**/**G1** observed using a POM. Open and closed circles show fan-shaped textures and dark fields in the Sm phase, respectively. Open and closed triangles show schlieren textures and dark fields in the N phase, respectively.

The relationship between γ_S_ and textures of **TM**, **R1**, and **R2** sandwiched between substrates pairs is shown in Figure 6. Homeotropic alignments were induced on relatively high γ_S_ surfaces for **TM**, on relatively low or high γ_S_ surfaces for **R1**, and on relatively low γ_S_ surfaces for **R2**. Since **R1** and **R2** are N and **TM** is Sm, the mechanism of phase transition is thought to be different. However, these homeotropic alignments were all considered to be derived from polar or nonpolar interactions between a molecular termination and substrate surface in this situation.

### 3.4 Characteristics of TM/DAN Mixtures on Chemically Treated Glass Substrates

**Figure 7:**
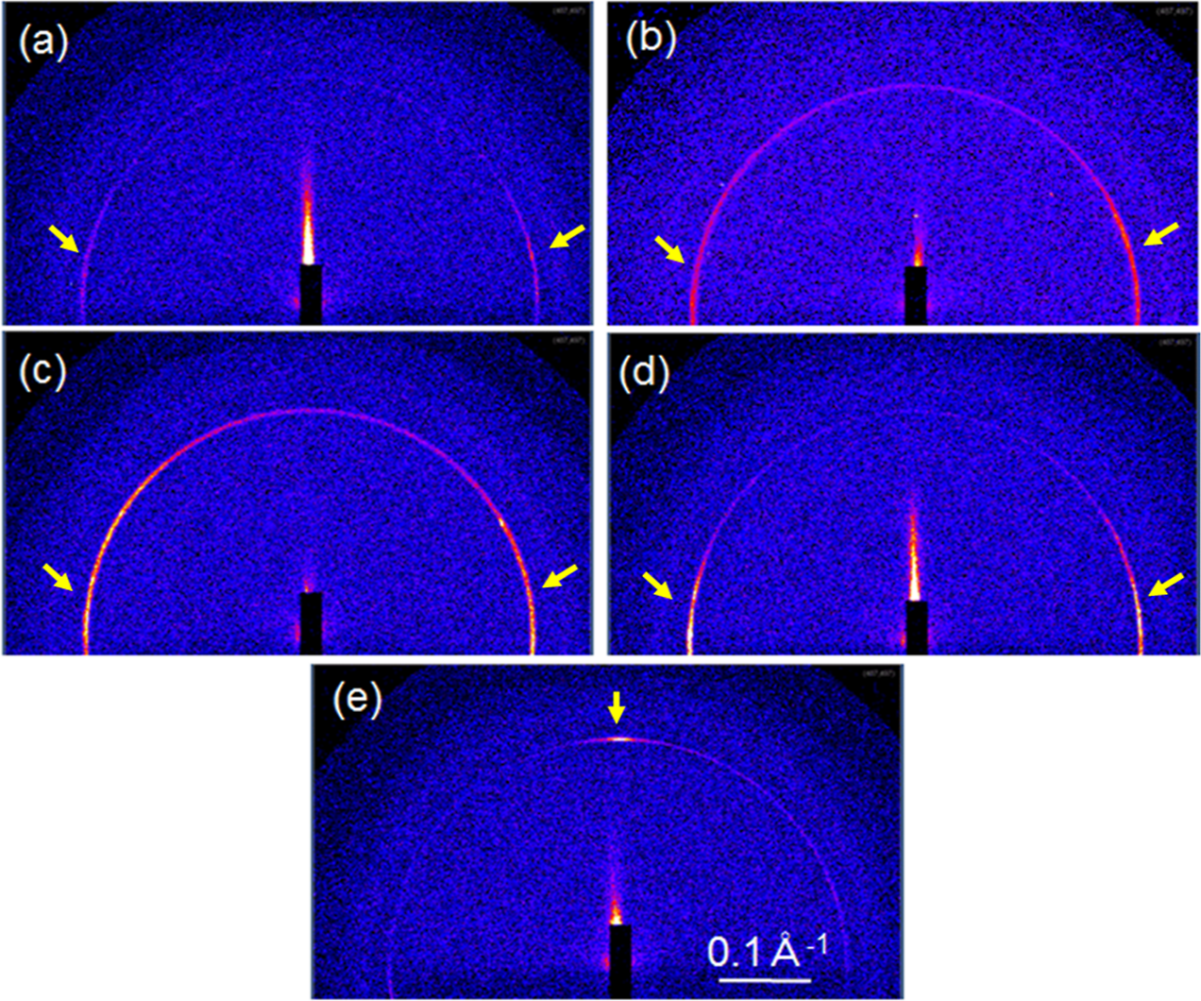
GISAXS patterns for **TM**/**DAN** droplets cured on (a) **C1**/**G1**, (b) **C2**/**G1**, (c) **C3**/**G1**, (d) **C4**/**G1**, and (e) **C5**/**G1**. Yellow arrows indicate diffraction peaks corresponding to the smectic layers.

**Figure 8:**
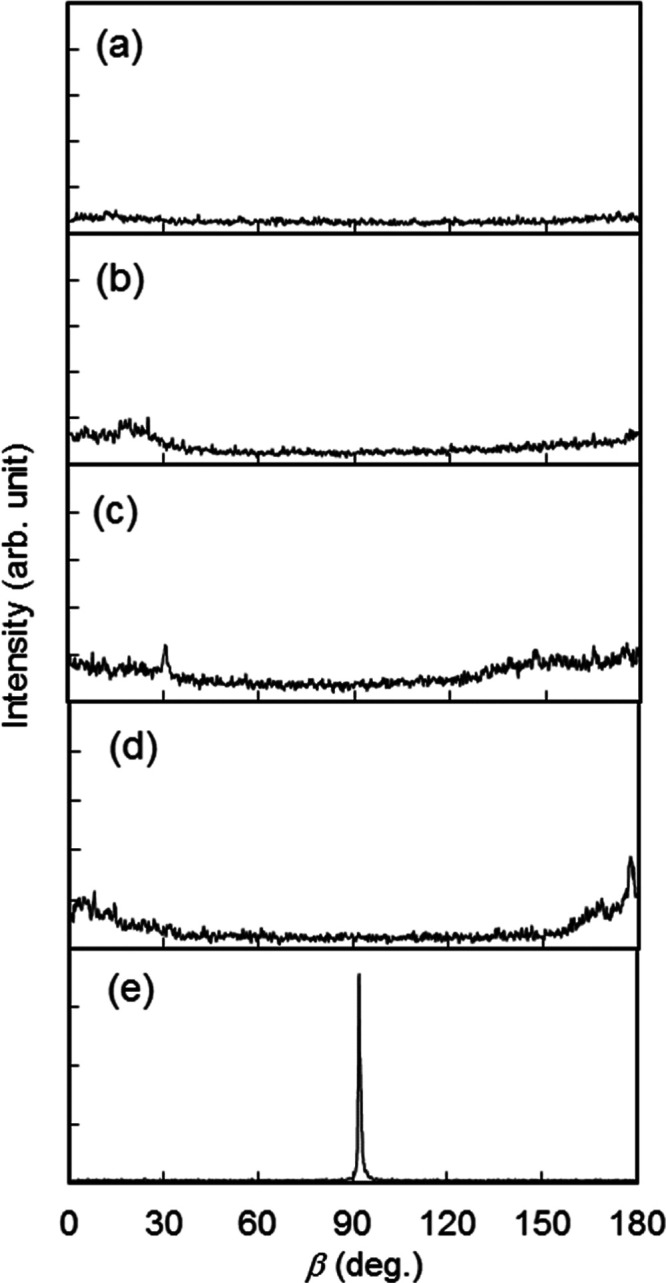
β scans of GISAXS intensities for **TM**/**DAN** droplets cured on (a) **C1**/**G1**, (b) **C2**/**G1**, (c) **C3**/**G1**, (d) **C4**/**G1**, and (e) **C5**/**G1**.

The surface effects on the molecular orientations of **TM**/**DAN** droplets cured on **C1**/**G1**, **C2**/**G1**, **C3**/**G1**, **C4**/**G1**, and **C5**/**G1** were investigated using GISAXS measurements. The evident spots in the GISAXS patterns of the **TM**/**DAN** droplets cured on **C5**/**G1**, which corresponded to Sm layers with a periodicity of approximately 22 Å in the vertical direction to **C5**/**G1**, were observed (Figure 7e). Whereas a half-ring appeared in the GISAXS patterns of the **TM**/**DAN** droplets cured on **C1**/**G1**, **C2**/**G1**, **C3**/**G1**, and **C4**/**G1**. The half-ring was slighter in the vertical direction to the substrates than that being in the transverse direction (Figure 7a–d). This was also obvious compared with the GISAXS intensity of β scans, as shown in Figure 8. These results indicate that **TM**/**DAN** is more likely to form the homeotropically aligned SmA domains on the **C5**/**G1** surface modified with amino groups, however, its surface had low γ_S_. This homeotropic alignment formation on **C5**/**G1** is considered to be derived from the reaction between the amino group on the surface and the epoxy group of **TM**.

The affinity between **TM** and **C5/G1** is thought to be lower than that between **TM/DAN** and **C5/G1** because amine is not included in **TM**. Also, the temperature of POM observations of 130 °C was too low to react between an epoxide of **TM** and an amino group on **C5**. For these reasons, it is considered that **TM** alone sandwiched between pairs of **C5**/**G1** did not form a homeotropic alignment (Figure 5f).

**Figure 9:**
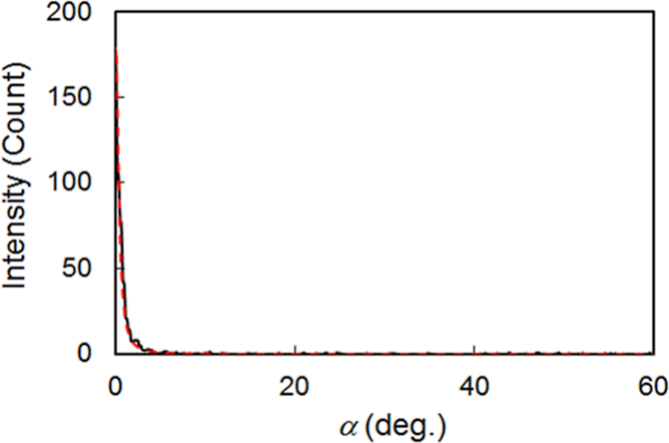
GISAXS intensities depending on angle α for homeotropically aligned **TM**/**DAN** mixture cured on **C5/G1**. Black solid and red dash lines indicate experimental results and Lorentzian fitting curves, *I*(α) = *h*/(1 + (α – *u*)^2^/*w*^2^) + *b*, which were calculated using solver function in Microsoft Excel. Experimental results of maximum intensities, angles at maximum intensities, full width at half-maximum, and background intensities were assigned to initial values of *h*, *u*, *w*, and *b* for the calculations, respectively.

**Figure 10:**
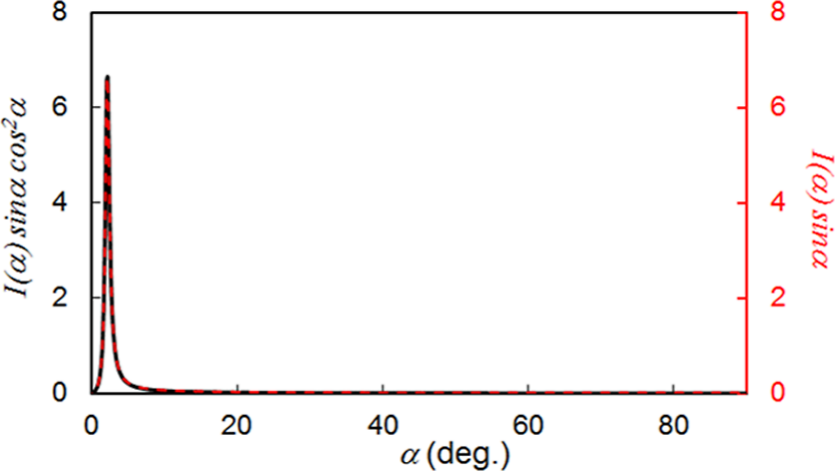
Graphical exhibitions of *I*(α) sin α cos^2^ α and *I*(α) sin α to determine orientational order parameter for homeotropically aligned **TM/DAN** mixtures cured on **C5/G1**.

The *S* of the induced homeotropically aligned Sm layers in the **TM**/**DAN** droplets cured on **C5**/**G1** was calculated from the β scan of the GISAXS pattern (Figure 8e) using eq 3, referring to calculations by Benicewicz et al.^29^ and Li et al.^30^
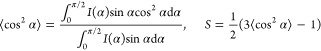
3where *I* and α express the SAXS intensity and angle between the Sm layer plane and the substrate surface, respectively. The α can be calculated from cos α = cos χ cos θ, where θ is the Bragg angle for the scattering and χ is β + 90°. Then, *I*(α) was obtained by fitting with the Lorentzian function (Figure 9); the graphs using the calculations are shown in Figure 10 for further information. As a result, the orientation parameter was estimated to be 0.95 for the homeotropically aligned **TM**/**DAN** droplets cured on **C5**/**G1**. This value was remarkable, and it was superior to 0.73 and 0.75 for the homeotropically aligned **TM**/**DAN** cured on untreated or UV-treated glass substrates.^18^ Moreover, the orientational order parameter of **TM**/**DAN** was expected to further increase when **TM**/**DAN** was sandwiched between a pair of **C5**/**G1** but not a droplet on the substrate because of no interfaces between the **TM**/**DAN** droplet and air that may generate disorder.

## 4 Conclusions

The relationship between the molecular orientation of a LCER and the functional groups on a glass surface modified using chemical surface treatments was investigated. A LC epoxy monomer **TM** was revealed not to form homeotropic alignment on a chemically treated substrate surface because of its relatively low γ_S_. However, a **TM**/**DAN** mixture formed a homeotropically aligned SmA structure by curing on a substrate surface modified with amino groups. This formation of homeotropic alignment was considered due to the attribution of the reaction between the amino group on the surface and the epoxy group of **TM**. The homeotropic alignment had a relatively high orientation parameter of 0.95. Therefore, it is expected to possess high thermal conductivity and be applied as high thermal conductivity adhesives or packaging materials for electrical and electronic devices. In addition, a homeotropic SmA LCER is also expected to be developed as a transparent high-thermal-conductivity material because homeotropic SmA LCs are known to be highly transparent.^47^
